# Perspectives and Outcomes of the Activity of a Reference Laboratory for Brucellosis

**DOI:** 10.3389/fvets.2017.00234

**Published:** 2018-01-05

**Authors:** Menachem Banai, Rita Itin, Svetlana Bardenstein

**Affiliations:** ^1^Department of Bacteriology, Kimron Veterinary Institute, Bet Dagan, Israel

**Keywords:** *Brucella*, bacteriology, serology, reference laboratory, lipopolysaccharide

## Abstract

One health is an emerging conceptual approach geared to harmonize the activities of the public health, veterinary services, and extension services within a single operative structure. Brucellosis is an important zoonosis worldwide, mostly involving nomadic populations but may often affect transboundary animal management and exotic domesticated animal farming such as camels and buffalo. Here, we provide contemporary knowledge on the disease and its causative agent, a Gram-negative bacteria belonging to the genus *Brucella*. Further, because of the zoonotic importance, we emphasize the need to assign a national reference laboratory for the disease and discuss how this would integrate into a “One Health” system. *Brucella* vaccines are live attenuated strains possessing the smooth phenotype, and vaccination, therefore, hampers the ability to maintain a national surveillance program due to concerns regarding the false positive vaccine-induced responses. In order to overcome these failings, we developed a combined approach based on rapid screening of mass numbers of serum samples by the fluorescence polarization assay, a cost-effective and accurate method, and confirmation of the true positive reactors by the complement fixation test, a highly specific method that is less sensitive to vaccine-induced antibodies. We demonstrate how, despite the high vaccination coverage of the small ruminant population in Israel, our results proved to be effective in discriminating between vaccinated and infected animals. The speed and accuracy of the method further justified immediate declaration of 37% of flocks as cleansed from brucellosis, thus reducing the burden of repeated tests among this population.

## Introduction

Brucellosis is one of the few severe zoonoses with worldwide distribution (1). The disease is associated with domestic animals, which play important roles in the dairy and meat industries (2) and is disseminated to the human population as a family or tribe cluster infection (3). Awareness of the association of the disease with domestic animals has raised in 1887 when Sir Bruce first identified the organism in pathological human samples, followed by implementation of serological diagnosis and *Brucella* isolation diagnostic approaches, respectively (4). To date, despite a century of learning, the disease still prevails at high rates in countries of the Latin America, South East Asia, the Middle East, and Persian Gulf (1).

Until the late 1960s, the international community recognized only six *Brucella* species based upon their unique association with a natural host in which they cause abortion in the last trimester of pregnancy (5). Four of these were considered zoonotic: *Brucella abortus* infecting cattle, but found associated with camelids and other bovids, *Brucella melitensis* infecting small ruminants but posing risk to bovids and camelids, and *Brucella suis* and *Brucella canis* infecting suids and canids, respectively. Two others have been recognized as non-zoonotic *Brucella* species: *Brucella ovis*, associated with orchitis and epididymis in rams and *Brucella neotomae*, infecting wood rats. Interestingly, recent studies have identified two potentially zoonotic sea mammalian species: *Brucella ceti* and *Brucella pinnipedialis*, associated with cetacean and pinniped brucellosis, respectively (6, 7). Molecular studies further showed that the genomes of the six-species, later conceived “Classical *Brucella*,” were highly homologous, justifying their unification into one single species, *B. melitensis*, upon which their sub-taxon being determined according to morphological and biochemical characteristics (8).

The discovery of two more classical *Brucella* species isolated from vole and red fox or soil, e.g., *Brucella microti*, endowed with higher metabolic activity (9, 10) and the *Brucella papionis* baboon-associated strain (11), further extended with the addition of the atypical strains belonging to the more basal lineages, e.g., *Brucella inopinata* BO1 (12) and BO2 human pathogens, and the red fox *Brucella vulpis* (13) or the motile frog isolates, has opened the door to widening the genus structure.

Most *Brucella* species have two chromosomes, of roughly 2.1 and 1.2 Mbp, ranging at 57.8% GC content (14). *Brucella* species lack common virulence factors such as motility, plasmids, and exotoxins. In contrast, they are equipped with a complete *virB* operon that endows them with an active type IV secretion mechanism (15) and a sheathed flagellum (16) found active in the basal frog lineages (17). The *Brucella* cell envelope contains lipopolysaccharide (LPS), a molecule shared by all Gram-negative bacteria. Due to a non-canonical structure, its lipid-A conveys a stealthy infection following the development of a poor innate immune response (18). The role *Brucella* LPS plays in the process of *Brucella* trafficking into the replicative niche of *Brucella-*containing vacuoles is currently debated (19). In contrast, its role as a major humoral stimulating antigen has laid out the structure for the serological diagnosis of brucellosis.

The epidemiology of brucellosis in Israel underwent significant changes throughout the years, affected mainly by the socioeconomic and geopolitical restrains in the region. During the 1950s and 1960s, *B. abortus* prevailed in the country. The disease was eradicated in 1985 following implementation of a “test and cull” policy combined with a full dose, sub-cutaneous S19 vaccination of replacement females. Later on, the increase in the small ruminant population and market demands eventually led to the emergence of *B. melitensis* in the country. A control program was then instigated in the 1990s based upon Complement Fixation Test (CFT) serological surveys of the adult population and implementation of a combined “test and cull” and live Rev. 1 vaccination program (20). Due to the lack of sustainable financing, the program was ceased in 1997 and the disease re-emerged in the beginning of the 21st century, leading to a record number of new human cases as well as the infection of a large dairy cattle farm in the southern region of Israel (Negev) (21).

## The Concept of a Reference Laboratory for Brucellosis

The “One health” approach addresses the multi-disciplinary facets of disease complexity involving livestock biosecurity, environmental conditions, veterinary, and medical extension services, as well as farm to fork aspects. Setting up a national reference laboratory complements the “One Health” concept in providing a centralization center with expertise on the standardization of methods corresponding with epidemiology, diagnosis, and human treatment, as well as implementation of prophylaxis and control programs in the livestock population. The laboratory should then focus its activity on strain isolation and typing (gold standard test) and characterization of serological tests that confirm exposure and/or infection among livestock populations and humans, respectively.

## *Brucella* Typing

In recent years, a return to a nomen-species structure of genus *Brucella* has gained support (22). In the laboratory, new isolates are first characterized according to their susceptibility to Fuchsin and Thionin dyes, as well as growth dependence on an enhanced CO_2_ atmosphere, and H_2_S and urease production. Staining by the modified Ziehl–Neelsen method is a rapid supplementary technique applied to confirm the disease at point of care sites based upon characteristic cell morphology and staining of *Brucella* species (23). In past years, *Brucella* metabolism was analyzed by the old oxygen consumption test, but this test has recently been replaced by a robust microplate metabolome method (24). Use of phage typing has remained supplementary to the methods, but the availability of different sources of phage variants complicate standardization of such analyses (25). Unfortunately, access to brucellaphage seed stocks has become rare and this situation has been worsened by the lack of knowhow on brucellaphage propagation and preparation of master routine test dilutions of phage suspensions emphasizing the need for harmonization and unification of a worldwide *Brucella* typing and classification system.

## *Brucella* Vaccines

Only a few *Brucella* vaccines have been approved for use in the field, all based upon live attenuated strains (26). Live vaccines are superior to killed or acellular vaccines as they survive in the host for a sufficient period of time required to induce a strong immune response by continuously challenging the host immune system. It was estimated that a good vaccine strain must survive for a minimum of 7.9 ± 1.2 weeks before clearance of the organisms by the immune system takes place. In contrast, virulent vaccine strains survive longer in the host, ruling out their use as suitable vaccines. The vaccine proficiency of the strain is established as inducing host cellular immune response active in clearance of a challenge strain (27) in comparison to an un-vaccinated control animal (28, 29). In contrast, the humoral response plays mainly as a secondary function in conveying protective immunity during early dissemination of the pathogen in the blood (30). Unfortunately, antibodies elicited by the vaccine interfere with surveillance and monitoring programs, due to the lack of effective DIVA methods that could distinguish between vaccine and field strain serology. Reference laboratories must play a role in determining vaccine qualities, establishing standard seed stocks and confirming vaccine lot qualities prior to vaccination/usage (20).

*Brucella abortus* S19/B19 and *B. melitensis* Rev. 1 have been established as official live attenuated reference vaccine strains for cattle and small ruminants, respectively. In recent years, *B. abortus* RB51 has been endorsed as a compromised vaccine fulfilling the protective activity against *B. abortus* in cattle without eliciting conflicting smooth antibodies that hamper serological testing (31). Readers are referred to a comprehensive review on the evolutionary adaptation of *Brucella* species to their hosts and how vaccination may intervene with the emergence of novel *Brucella* (4). Further reading is recommended regarding vaccine efficacy and risks associated with Rev.1 and S19 implementation at different conditions (26, 32).

## Molecular Typing

*Brucella* typing and classification can now be achieved by molecular approaches. DNA amplification of a specific sequence by PCR is a basic approach in bacteriology aimed at gene cloning, on the one hand, and gene characterization on the other and is currently widely used in diagnosis (33). AMOS PCR (standing for *B. abortus, B. melitensis, B. ovis*, and *B. suis*) was established in the 1990s as a primary method of classification of these species. The method is based on the IS*711* sequence inserted at different allelic sites in the chromosomes of the different species, producing characteristic amplified fragments of the target sites. Among *B. abortus*, AMOS PCR is limited to bvs. 1, 2, and 4, inferring insufficient sensitivity of this method to other *B. abortus* biovars (34, 35). Several other PCR-based molecular methods have been described in the literature culminating to the approval of the Bruce-ladder PCR by the OIE as a recommended approach (36).

Variable number tandem repeats (VNTR) that is also referred to as multiple loci VNTR analysis, is a molecular amplification method of micro- and macro-satellite DNA fragments that depict specific molecular fingerprints associated with an epidemiological strain. The method targets 16 molecular sites on the *Brucella* chromosome, divided into eight Panel A markers that specifically distinguish between *Brucella* species and 5 and 3 Panel B hypervariable epidemiological genotypes, respectively (37). Although effective in defining epidemiological linkages among isolates, this method suffers a major flaw by including multiple *Brucella* biovars within a single genotype.

## High-Throughput Molecular Typing

Whole genome sequencing has been instigated in recent years in genomic characterization of bacteria. At the chromosomal level, single-nucleotide polymorphism plays an important tool in identifying phylogenetic linkages. This method has been widely implemented in describing *Brucella* phylogenetic linkages as well as associating novel strains into genus *Brucella*. Development of a national bank of a strain collection helps in showing global trends of *Brucella* spread or clonal evolution for which multi-locus-sequence analysis is considered a favored approach (36).

## Serological Tests

*Brucella* species cross-react among each other as well as with *Yersinia enterocolitica* O9 and other Gram-negative bacteria (38). Furthermore, disease first progresses with the development of IgM antibodies and further succeeds by the development of IgG antibodies during the chronic state or alongside pathogen persistence in the animal (39). Finally, antibody isotypes might vary upon the challenge dose, the site of infection, and the capacity to establish bacteremia or develop a limited local infection in a specific organ or tissue, as well as antibiotic treatment. Therefore, these factors must be taken into account when designing a serological approach in the laboratory.

A reciprocal correlation exists between a test’s sensitivity and its specificity. Sensitivity is increased at the expense of increased background noise, therefore, reducing specificity. In contrast, specificity is increased by reducing background noise and cross reactivity with heterologous antibodies at the expense of reduced sensitivity. IgM antibodies are pentamers, which promote the development of a net between antibodies and cells, thus causing agglutination in the tube [serum agglutination test (SAT)]. In contrast, IgG antibodies interact with the host’s complement fixing system. Such a function forms the basis of the CFT, a lytic readout response against targeted antigens. Because IgM and IgG antibodies rise at different stages of the disease, agglutination and CFT complement each other in diagnosis, the first highlighting on acute infection whereas the latter indicates a chronic persisting infection, respectively. Thus, CFT provides a higher predictive value than SAT and, as such, has been affirmed by OIE as the prescribed method for animal trade between countries.

The enzyme-linked immunosorbent assay (ELISA) and fluorescence polarization assay (FPA) are two high-throughput serological techniques aimed at detecting serum antibodies (analyte) in a given sample. Because the two systems employ different underlying principles, their performance by sensitivity and specificity may vary. Two ELISA methods have been validated, indirect ELISA that measures binding of secondary antibodies to a primary antibody isotype bound onto the *Brucella* LPS antigen, and competitive ELISA, which measures the competitive binding between anti-*Brucella* LPS monoclonal antibodies and host’s antibody onto the same reactive site. Both ELISA methods suffer from having non-specific binding of conjugate to background substrates and as such, their reading signal is increased. To avoid background noise, blocking and washing steps are introduced as intermediated steps in the method, thus reducing analyte concentration in the system and reducing the test’s sensitivity.

Unlike ELISA, FPA measures antibody (analyte) binding to a soluble antigen by a homogenous measurement method without interventional steps such as blocking and washings. Because of the principle of homogeneity, this method measures the interaction of all participating antibody isotypes in the sample with the soluble antigen, establishing the highest final reading. It is considered, therefore, highly sensitive. Test specificity has been similarly achieved due to using the soluble *Brucella* O-PS antigen, which omits Lipid A and core LPS epitopes in the reaction mixture. The method thus achieves a maximum performance value of close to 200%, surpassing ELISA as the best performing technique (40).

Plate agglutination methods, such as Rose Bengal Test (RBT), resemble FPA as homogenous methods. Unlike FPA, RBT is performed manually, limiting the number of samples a laboratory can process per day. RBT is considered, therefore, a screening approach surpassed by the high-throughput ELISA and FPA methods, however, because of its low price, some laboratories rather use this test as an alternative test to CFT.

The establishment of an international standard serum, which aimed to internationally harmonize test results, was considered by the OIE, leading to development of the second International standard anti-*Brucella abortus* Serum (second ISABS) arbitrarily assigned with 1,000 IU for SAT and CFT tests, respectively (23). Local serum standards could thus be established in different countries, allowing for the first time, the comparison of serological tests among different countries. A similar serum standard against *B. melitensis* infection in the small ruminant population has recently been described (41). Nonetheless, neither standard sera directly correlate with the diagnosis of human brucellosis.

## Decision-Making

Small ruminant population in Israel includes intensively managed flocks grown in rural places for dairy and meat production and extensively managed open flocks often pasteurizing on agricultural lands at southern Israel (Negev), and less at the northern part of the country. Human brucellosis is mostly associated with the nomadic animals due to close contacts with the animals. Following the 1990s’ campaign (20), implementation of a full dose, ocular Rev. 1 vaccination of the young replacement females was enforced in the country. In spite of the vaccination program, due to cessation of the national control program, a new burst of human cases rose, most occurring in the Negev, leading to a public outcry to instigate a new national campaign.

The new program targeted the Negev, covering about 250 pasteurizing flocks (some 15,000 animals) in close proximity to dairy cattle herds, and others of risk to humans, including more than 1,000 flocks (over 250,000 heads) respectively.

In order to overcome DIVA concerns, we chose FPA as a screening method (being rapid and highly sensitive), and CFT [omitting most vaccine reacting individuals from the population (42)] as a confirmative method.

## Results and Conclusion

For statistical purposes, we chose sampling data that were available from the second cycle of the brucellosis campaign. At this stage, we expected most of the population to be tested negative by FPA as most infected animals have already been excluded from the population. Other FPA results could have been distributed between FPA positive (FPAp) and FPA suspect (FPAs) reactors, respectively. Among these reactors, we anticipated that FPAs reactors would belong to the vaccinated population expected to be found CFT negative (CFTn). We chose a free “QuickCalcs” calculator, which compares agreement between two methods in terms of a Kappa value, based upon the number of selected categories in the system. Comparison was conducted upon the observations obtained from the two population categories, FPAp:CFTp and FPAs:CFTn, respectively. As shown in Table 1, a total of 4,684 animals were tested by the FPA method revealing 1,101 (23.5%) of FPAp and FPAs responders in total that further were distributed within groups as follows: FPAp:CFTp (97, 8.8%), FPAs:CFTp (10, 0.9%), FPAp:CFTn (269, 24.4%), and FPAs:CFTn (725, 65.8%), respectively. As shown in Table 1, the inter-rater qualitative agreement by Cohen’s Kapp test indicated fair agreement between FPA and CFT tests, with Kappa coefficient of 0.306 (95% CI 0.253–0.358).

**Table 1:** Calculation of the agreement between fluorescence polarization assay (FPA) and Complement Fixation Test (CFT) observations by the Cohen’s Kappa test.

	FPA positive	FPA susceptible	Total
CFT positive	97	10	107
CFT negative	269	725	994
Total	366	735	1,101

*Number of observed agreements: 822 (74.66% of the observations)*.

*Number of agreements expected by chance: 699.1 (63.50% of the observations)*.

*Kappa = 0.306*.

*SE of kappa = 0.027*.

*95% confidence interval: from 0.253 to 0.358*.

The strength of agreement is considered to be “fair.”

As seen in Table 2, we tested 43,756 animals representing 760 flocks during the first cycle of the campaign. FPAs (3,179) and FPAp (3,066) comprised approximately 14.3% of the population. At the flock category, a total of 482 (63.2%) flocks were suspect/positive by FPA, 203 (26.7%) comprised true CFTp and 279 (36.7%) were CFTn, respectively, allowing omission of the latter group from future monitoring and thus reducing the burden on the laboratory. This highlights on the advantage of using FPA on a wide scale surveillance campaign in a population with a high vaccine coverage, first as a screening approach, using CFT as a confirmation method, and then, as a sole method during the finalization of the eradication campaign. In conclusion, our work confirms the feasibility of using FPA in screening and eradication of vaccinated flocks in other places worldwide.

**Table 2:** Comparison of numbers of responders between fluorescence polarization assay (FPA) and Complement Fixation Test (CFT) among serum samples randomly taken at early stages of the 2016 campaign.

	FPA positive	FPA suspect	FPAn
	3,066	3,179	37,511
CFTp	2,255 (36.1%)		
CFT	3,990 (63.9%)		

*P, positive; N, negative; S, suspected*.

## Ethics Statement

The study was exempt from one or more of the above requirements because it was conducted for the purpose of the control/eradication of brucellosis during a national campaign.

## Author Contributions

MB conceived the project and wrote the manuscript. SB produced the data and RI assisted in performing the tests and data organization.

## Conflict of Interest Statement

The authors declare that the research was conducted in the absence of any commercial or financial relationships that could be construed as a potential conflict of interest. The handling editor is currently co-organizing a research topic with one of the authors MB and confirms the absence of any other collaboration.
